# Decision discovery using clinical decision support system decision log data for supporting the nurse decision-making process

**DOI:** 10.1186/s12911-024-02486-3

**Published:** 2024-04-18

**Authors:** Matthijs Berkhout, Koen Smit, Johan Versendaal

**Affiliations:** 1 Digital Ethics, HU University of Applied Sciences Utrecht, Heidelberglaan 15, Utrecht, 3584 CS The Netherlands; 2 Open University of the Netherlands, Valkenburgerweg 177, Heerlen, 6419 AT The Netherlands

**Keywords:** Decision management, CDSS, Decision mining, DMN, Discovery, Decision-making

## Abstract

**Background:**

Decision-making in healthcare is increasingly complex; notably in hospital environments where the information density is high, e.g., emergency departments, oncology departments, and psychiatry departments. This study aims to discover decisions from logged data to improve the decision-making process.

**Methods:**

The Design Science Research Methodology (DSRM) was chosen to design an artifact (algorithm) for the discovery and visualization of decisions. The DSRM’s different activities are explained, from the definition of the problem to the evaluation of the artifact. During the design and development activities, the algorithm itself is created. During the demonstration and evaluation activities, the algorithm was tested with an authentic synthetic dataset.

**Results:**

The results show the design and simulation of an algorithm for the discovery and visualization of decisions. A fuzzy classifier algorithm was adapted for (1) discovering decisions from a decision log and (2) visualizing the decisions using the Decision Model and Notation standard.

**Conclusions:**

In this paper, we show that decisions can be discovered from a decision log and visualized for the improvement of the decision-making process of healthcare professionals or to support the periodic evaluation of protocols and guidelines.

**Supplementary Information:**

The online version contains supplementary material available at 10.1186/s12911-024-02486-3.

## Background

Decision making in healthcare is complex and increasing in complexity [1]; notably in hospital environments where the information density is high, e.g., emergency departments, oncology departments, and psychiatry departments [2–4]. For example, within the Intensive Care Unit (ICU), healthcare professionals are faced with numerous complex decisions every day, many of which are made under time pressure. Zavala et al. [5] stated that consultation time is limited for physicians, affecting their decision making. This can easily lead to errors and adverse events causing an increase in healthcare costs.

### Limitations of current systems

Protocols, hospital information systems, and (clinical) decision support systems (CDSSs) are in place to support the healthcare professional during their work [6]. CDSSs aid healthcare professionals with their decision-making process. The healthcare professional enters patient data into the CDSS, and the system advises on the best course of action [7]. A CDSS uses predefined rules to determine the best available option using relevant and available patient data. However, the growing number of CDSSs, monitoring systems, and predictive tools has not lowered inaccuracies in decision making [8, 9]. Notably, monitoring systems and CDSSs have increased the number of alarms to which a healthcare professional may be exposed to as many as 1000 alarms per day [10]. The majority (between 80 and 99%) of these alarms do not require immediate attention, resulting in a decreased response rate and a phenomenon experienced by healthcare providers described as ‘alarm fatigue’ [10, 11]. Alarm fatigue increases the clinician’s response time or decreases the response rate due to too many alarms and eventually causes them to be desensitized [12, 13]. Numerous attempts have been made to address both the deficiencies in existing decision support systems and the resulting alarm fatigue, often with the introduction of yet another new, innovative tool/application [8, 14]. Many of these developments fail to reach clinical implementation, fostering an increasing level of cynicism and ‘innovation fatigue’ instead [15, 16]. This is part of a bigger trend, stated by Granja et al. [17] that one of the barriers for implementation is the undefined role and change of work practice of the parties involved [17]. In addition, maintaining the knowledge within the CDSSs is time consuming, and thus costly, while it is critical for a successful implementation of a CDSS into the workflow of healthcare practitioners [18, 19]. It is clear that an alternative approach must be adopted focusing on the trend of AI and data analytics [20].

### Objective

Therefore, rather than adding a new system and/or more alarms and notifications into daily healthcare processes in hospitals, we aim to explore whether existing systems can be optimized by adding retrospective analysis of decision log files stored in existing hospital systems, e.g., the electronic health record, CDSSs, and hospital information systems. Furthermore, the aim is to improve the decisions, using retrospective data, without the burden of a new implementation or new system into the workflow of daily caregivers.

### Decisions in data

**Fig. 1:**
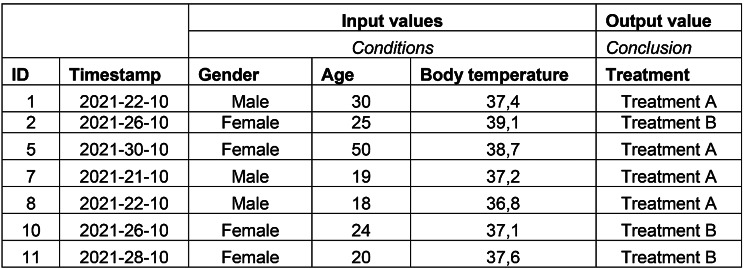
Example of a simple decision log

Decisions are logged daily into information systems or CDSSs and the decision logs can be exported from the information systems [21]. The decision logs provide a direct representation of the day-to-day decisions made by healthcare professionals. A decision log consists of a (trace) ID, a timestamp, a (set of) condition(s), and a conclusion. An example of a simple decision log is shown in Fig. 1. In this example, the decision log comprises a patient ID, a timestamp, and three input values, Gender, Age, and Body Temperature. These input values are the conditions on which a treatment, in this case the conclusion, is started; Treatment A or Treatment B.

To further elucidate the concept of decision logs, consider the following real-world example from an emergency department. The decision log captures critical data points such as patient ID, timestamp, presenting symptoms, vital signs, and the resulting treatment decision.

The Decision Model and Notation (DMN) standard can be employed to visualize and interpret this decision log [22]. DMN can also be employed to exchange and execute decisions in a CDSS. DMN allows for the graphical representation of the ‘*how’* of decision-making, thereby facilitating a more transparent and understandable decision-making mechanism. For instance, the decision to “Administer Nitroglycerin” for a patient with “Chest Pain” and a Blood Pressure value of “160/90” can be modeled in DMN to show the decision tree or table that led to this particular treatment choice. This enables healthcare professionals to better understand the rationale behind each decision, aids in the periodic evaluation of protocols and guidelines, and can help with the training of junior nurses and their clinical decision making. By integrating DMN with decision logs into CDSSs, healthcare organizations can achieve a more robust, transparent, and efficient decision-making process.

### Role of information technology

Because Information Technology is transforming hospitals and the way healthcare is carried out and documented, huge amounts of patient information is generated, exchanged, and stored about patients. This includes all data relating to all aspects of care e.g., diagnosis, consultation, medication, and laboratory results. This data could potentially become readily available at the bedside of the patient to support decision making using CDSSs [23]. Historical data gathered from patients can be leveraged to improve the CDSSs and provide feedback, which, in turn, improves the quality of decision making by healthcare professionals [24]. The decisions within logged data (decision logs) can be discovered using decision mining. With decision mining, organizations can identify decisions and discover trends that are recorded in information systems such as electronic health records or CDSSs. Several classification algorithms can be used for decision mining to analyze decision logs [25]. Classification algorithms are used to place data into preset categories, which is a form of pattern recognition [26]. There are different types of classification algorithms, e.g., Decision trees, fuzzy algorithms, Random Forest, and Artificial Neural Networks [27, 28]. While classification algorithms are not new, using them for the discovery of decisions for retrospective analysis is [21, 25, 29]. Some attempts were taken, especially in the field of process mining [30–32]. For example, Bazhenova et al. [25] explored the usage of a neurofuzzy classifier for discovering decisions from a process event log. However, this was based on artificial neural networks, a type of algorithm that is considered a black box, introducing a new set of challenges [25].

In this study, we use a fuzzy-only algorithm as a basis for discovering decisions. The characteristics of fuzzy algorithms are, (1) that it can deal with uncertainty, which could arise as a common characteristic of healthcare data. (2) It helps mimic the logic of the human thought and (3) is a flexible machine learning technique while being explainable [33].

Therefore, we aim to answer the following research question: *How can the fuzzy algorithm be adapted to discover business decisions and business logic in a healthcare context?*

The remainder of this paper is structured as follows: First, the methodology section in which we explain how we employed the design science research approach. Next, we present the algorithm and explain how it works. Furthermore, the algorithm is demonstrated using one case in the simulation section. This is followed by a discussion in which the practical and scientific implications of our study and the algorithm are considered. Finally, conclusions are drawn, and future research directions are posed in the last section.

## Methodology

To answer our research question, the Design Science Research Methodology (DSRM) was adopted for this study. DSRM is commonly used for wicked problems. As the healthcare system has modernized in the last decades, it has also become rich with complexity. It involves a series of moving parts with, e.g., different stakeholders, financial concerns, clinical standards, health protocols, and government regulations [34]. The complexity becomes even higher as every patient and every disease and underlying conditions comes with its own particularities that directly or indirectly influence each other. Therefore, we consider the DSRM approach in adapting the algorithm appropriate considering the wickedness of the underlying problem. Moreover, the artifact/algorithm we aim to construct/adapt will be novel, effective, and useful: another characteristic that justifies the DSRM approach. DSRM structures the development and validation of information system artifacts whilst requiring their relevance to be grounded in practice and their rigor based on the existing body of knowledge [35, 36]. This helps us structuring the development of the fuzzy algorithm. One of the main advantages of the DSRM is that it focuses on the iterative development of artifacts, in our case the fuzzy algorithm, so that it can be utilized in a healthcare context. To further guide the development of the fuzzy algorithm we used the seven DSRM guidelines proposed by Hevner et al. [36] As such DSRM guides the definition of our study protocol. These guidelines are converted into six Design science research activities and accompanying tasks, see Table 1 [36, 37].

**Table 1:** DSRM activities

DSRM activity	Tasks
1. Identify problem & motivate	Conducting interviews with experts from (IT) organizations, healthcare practitioners, and scientific researchers. This study is already presented in [25]
2. Define the objectives of a solution	Identify appropriate algorithms and requirements for decision mining discovery
3. Design and development	Design of artifact (algorithm for discovering decisions) for healthcare
4. Demonstration	For the demonstration, a realistic case is used. The case is based on determining if a patient needs a catheter. This case is extracted from the RCN guidelines [38]
5. Evaluation	In this paper, we show the feasibility of using an adapted fuzzy algorithm by evaluating the algorithm from a technical and theoretical viewpoint
6. Communication	Performed during the full duration of the project for which different outlets are selected, such as symposia, workshops, conferences, and journals. This way, practitioners as well as researchers are informed about the results of the project

Although this research project and its underlying objectives encompass all phases, activities, and tasks outlined in Table 1, the focus of the current study is specifically on the third and fourth activities. This targeted focus is deliberate for several reasons. First, this study is part of a broader Ph.D. research project, and as such, the other activities—namely activities one, two, five, and six—are discussed in detail in separate studies that have been published elsewhere. Second, by concentrating on the third and fourth activities, this paper aims to delve deeper into these specific aspects, providing a more detailed and nuanced understanding that would not be possible if all activities were covered in a single paper. Third, the choice to focus on these activities aligns with the project’s phased approach, allowing for a more structured and in-depth exploration of each component. The focus on these activities also gives the opportunity to adapt to the found results during the totality of the project. Finally, for the sake of completeness and to provide a coherent narrative, we discuss the other activities and their outcomes briefly. This ensures a comprehensive understanding of the research project, from its inception to its current state, thereby making the reasoning behind each development stage explicit.

### Activity one: identify problem & motivate

In order to set up the context for this research, semi-structured interviews with experts from IT organizations, healthcare practitioners, and scientific researchers in the field of study were performed as part of a pre-study. The objective was to understand the potential of data that is gathered in hospital information systems and CDSSs and to identify the problem(s) currently existing.

### Activity two: define the objectives of a solution

This activity aims to identify relevant algorithms by mapping gathered requirements to potential algorithms divided into three phases. The first phase consists of gathering requirements by conducting semi-structed interviews with researchers and data scientists working in a commercial environment who are familiar with data mining, process mining, decision mining, and AI. The second phase consists of identifying algorithms by conducting a literature review and conducting semi-structured interviews. The third and last phase consists of mapping the requirements against the algorithms by experts in the field of decision mining. The outcome of this study is presented in [25].

### Activity three: design and development

**Fig. 2:**
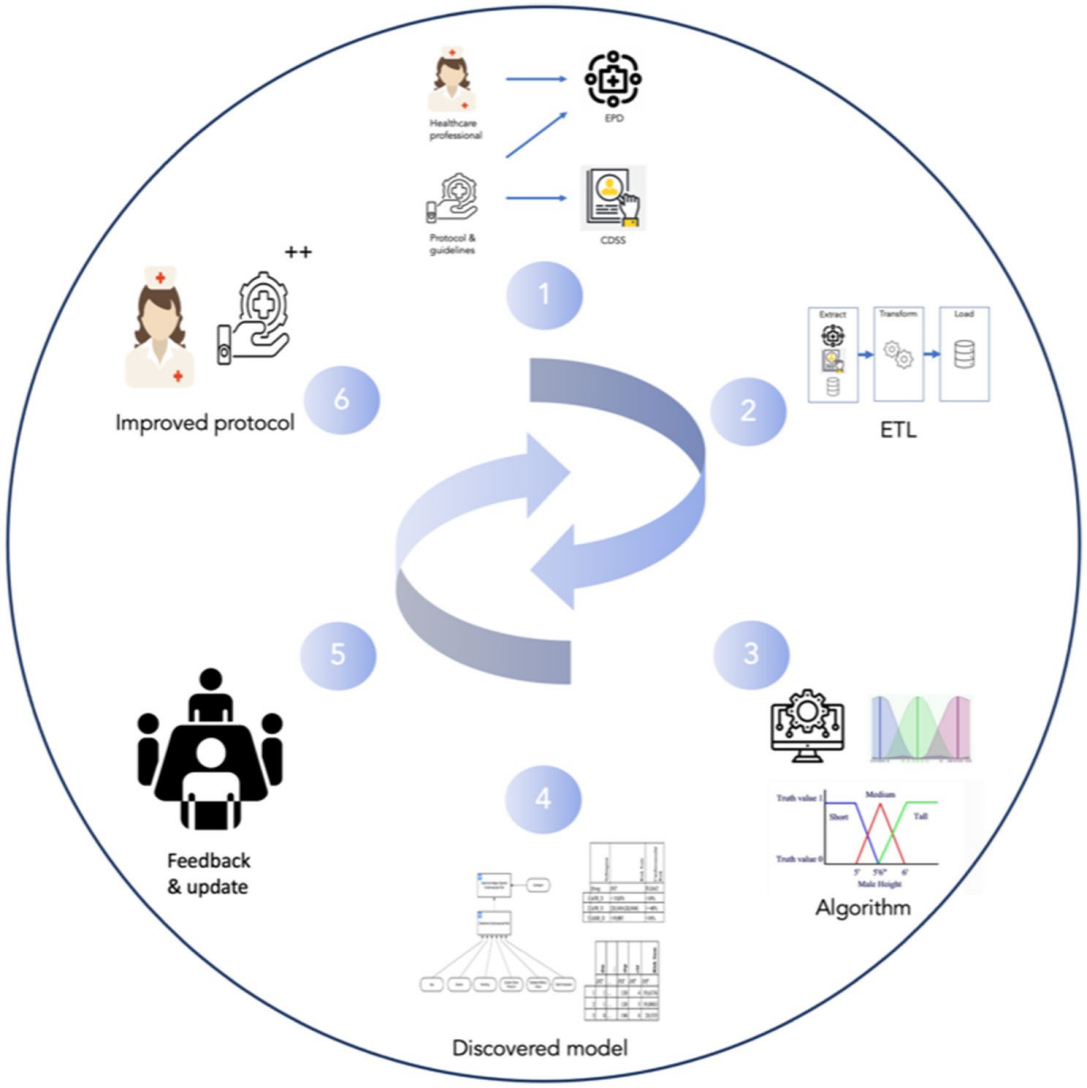
Process and usage of artifact

**Fig. 3:**
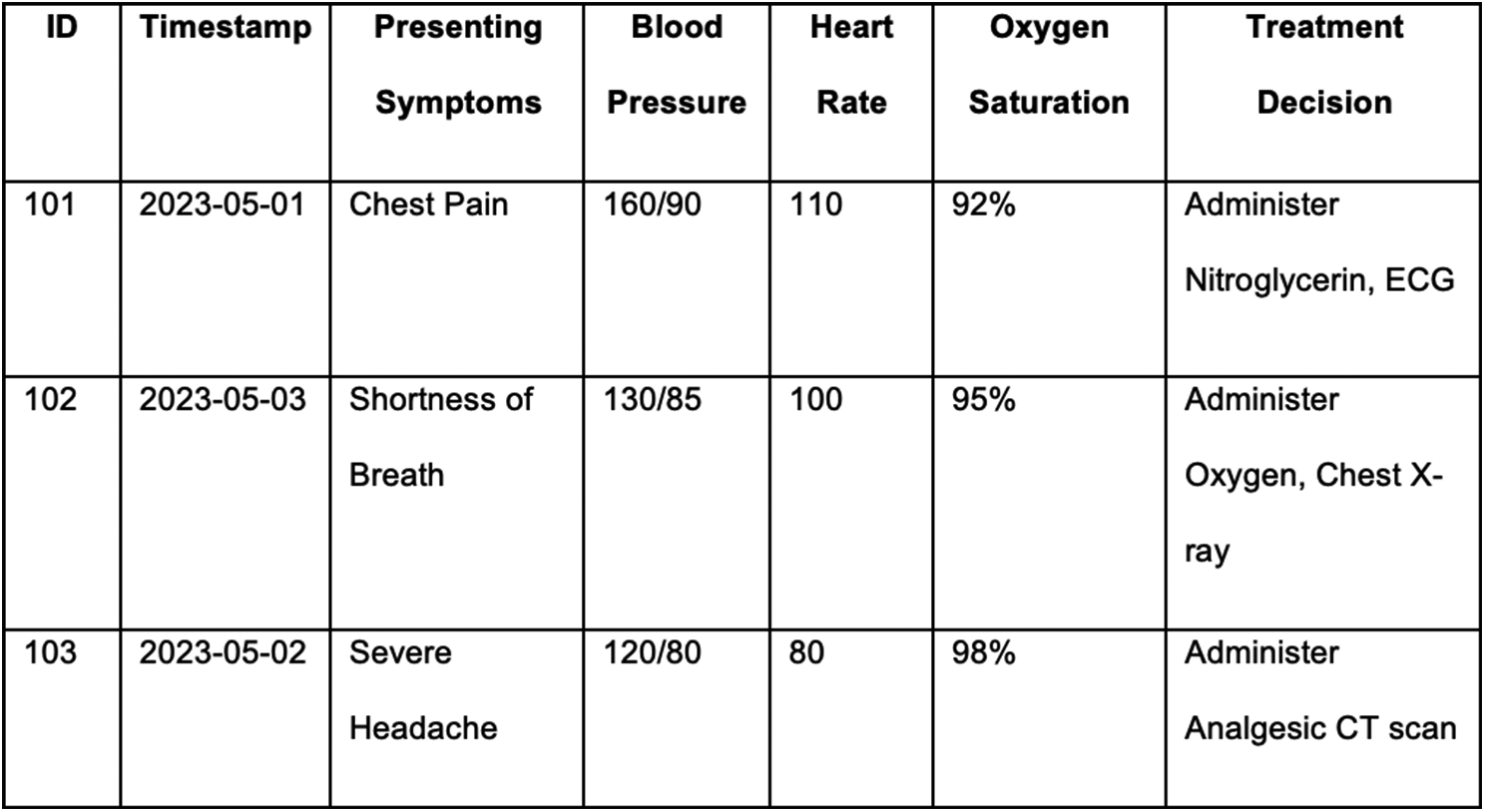
example of a decision log

In this phase, the algorithm will be designed and adapted to fit in the evaluation phase by healthcare practitioners. Therefore, the normal course of events within the evaluation process is changed as the algorithm that is designed will be added to the evaluation of made decisions or the evaluation of a protocol. The changes in the evaluation are divided into five steps, as seen in Fig. 2 : [1] Data coming from healthcare practitioners, protocols, and guidelines are stored in hospital information systems and EPDs [2]. The log files of such systems can be extracted and transformed into a decision log, fig. 3 [3]. The algorithm uses the decision log to analyze the data and [4] to visualize it into a human readable model, in this case using the Decision Model and Notation (DMN). DMN is the de facto standard for modeling and visualizing decisions. A further elaboration on using this standard can be found in the discussion [5]. The output models can be used as extra input for [6] improving the protocols and guidelines. These steps can be executed multiple times as part of an iterative improvement of a protocol.

### Activity four: demonstration

The algorithm must be validated after it has been designed. An experiment based on (1) synthetic or (2) real-life datasets is an appropriate research method to assess the effectiveness and applicability of a product, algorithm, method, framework, or category [39]. With synthetic datasets, the researchers can modify the model, the inputs, the experiment setting, and the actual simulation through experiments. The model and experiment design for real-life data can still be controlled, but less control over the input and simulation can be claimed. For both synthetic and real-world-based studies, reproducibility and traceability are essential needs [40]. In this study, we use a real-life decision, namely determine catheterization of a patient [41], but with artificially generated data, as there is a theoretical model available. The theoretical model is used to check whether the basic output of the algorithm is accurate and valid.

The following components for the experiment setup are reported: Overview of the basic model, model logic, scenario logic, algorithm, and applied components [42]. Finally, it is necessary to report on the following aspects of the experiment’s execution: Initialization, runtime, and estimation methods, based on the approach of [43].

### Activity five: evaluation

The evaluation phase commences following the algorithm’s demonstration. In this activity, synthetic data is employed to simulate a decision log, serving as a basis for evaluating the algorithm’s performance. The use of synthetic data is a strategic choice for several reasons. First, it allows for the creation of a controlled environment that mimics an authentic context, albeit with artificial content. This approach aligns with Wieringa’s framework [43], which identifies two types of generalizations when transitioning from research to practice: inductive generalization from small to large samples, and model-based generalization from experimental settings to real-world scenarios. In the next iteration of the algorithm, we also scale up to larger samples.

**Fig. 4:**
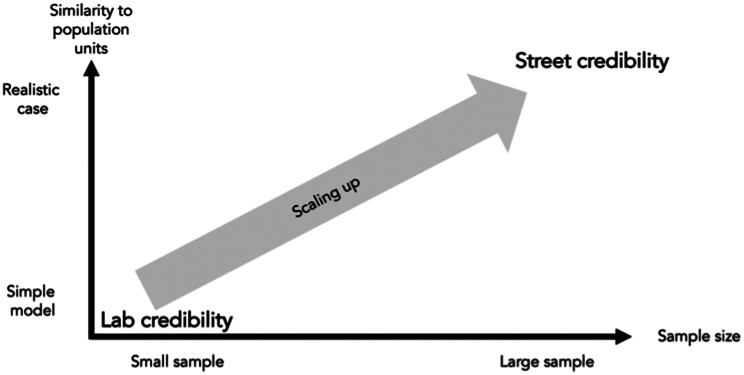
Scaling up evaluation [44]

In our study, we opt for a relatively small sample size to facilitate a more manageable and focused evaluation. While the case under study aims to be as realistic as possible, the use of synthetic data serves as a stand-in for real-world data. This method enables us to test the algorithm’s efficacy and reliability without the complexities and variabilities inherent in actual healthcare settings. This will be done in future iterations of designing the artifact, as mentioned by Hevner et al. [35]. Moreover, the synthetic data allows us to isolate specific variables and conditions, providing a clearer understanding of the algorithm’s performance and potential limitations [44]. As depicted in Fig. 4 , this approach provides a pathway for scaling up the findings to more complex, real-world cases in future research.

### Activity six: communication

This activity aims to disseminate the results. Throughout the duration of this research project, several activities were conducted. The activities consisted of oral presentations for physicians, nurses, nurse students, and, IT professionals. The poster presentation was held for people in the field of study. One paper is published in the proceedings of a peer-reviewed conference. This publication is also part of disseminating the results of this study.

## Algorithm design

In this section, we propose a methodology to discover and visualize multiple decisions extracted from an array of decision logs. The algorithm driving this methodology is a fuzzy classifier. The selection of this particular algorithm was not arbitrary; it was the outcome of an evaluation process involving multiple algorithms, done in an earlier study within this cycle [25]. The fuzzy classifier algorithm was one of the algorithms that could be used with a small adaptation by adding support for multiple decisions. Other studies already used a fuzzy algorithm to discover decisions, but they used a neurofuzzy classifier [30, 45]. In this study we do not use neural networks, because they are not favorable to use as neural networks are prone to overfitting, are seen as black boxes which lack transparency, and have a computational burden [46].

**Fig. 5:**
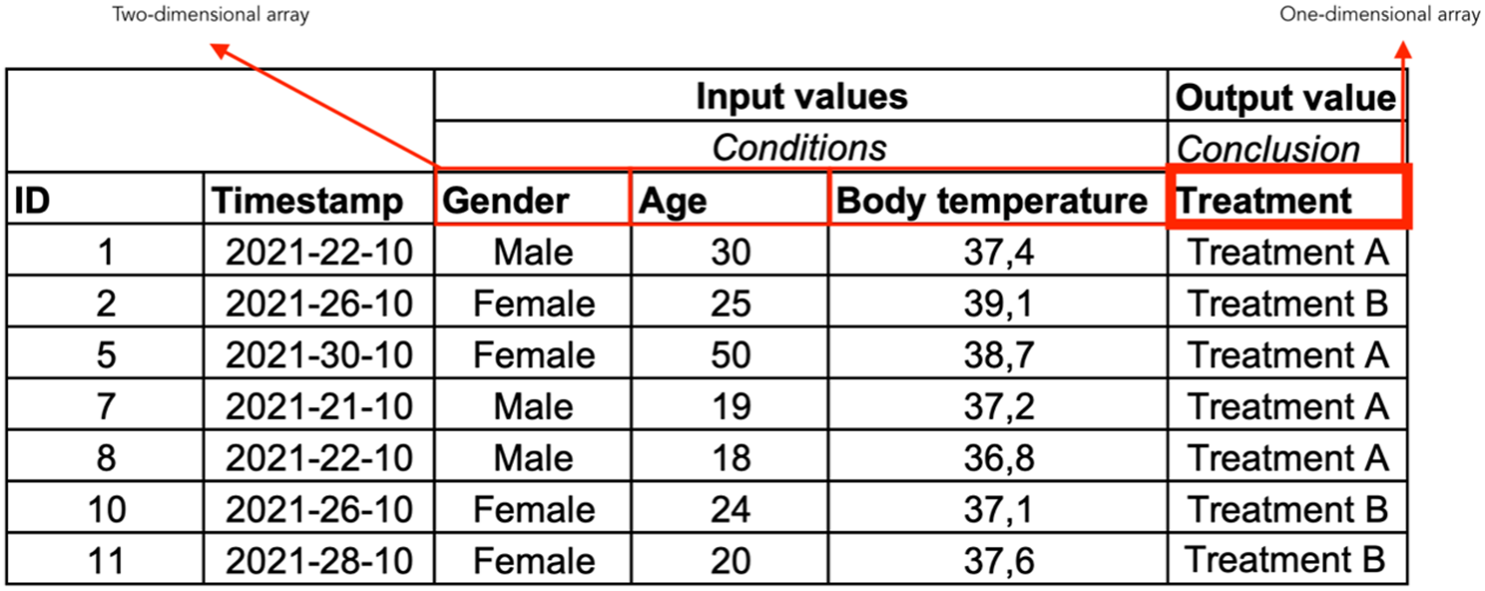
Dividing in arrays

The algorithm operates a dataset that is structured into columns with target values (output value) and input values. The target values are a one-dimensional array and are categorical. The input values are a two-dimensional array consisting of categorical or continuous columns, as seen in Fig. 5 .

When a column contains continuous values, the algorithm will walk through this column in a loop and start with the first data point

**Fig. 6:**
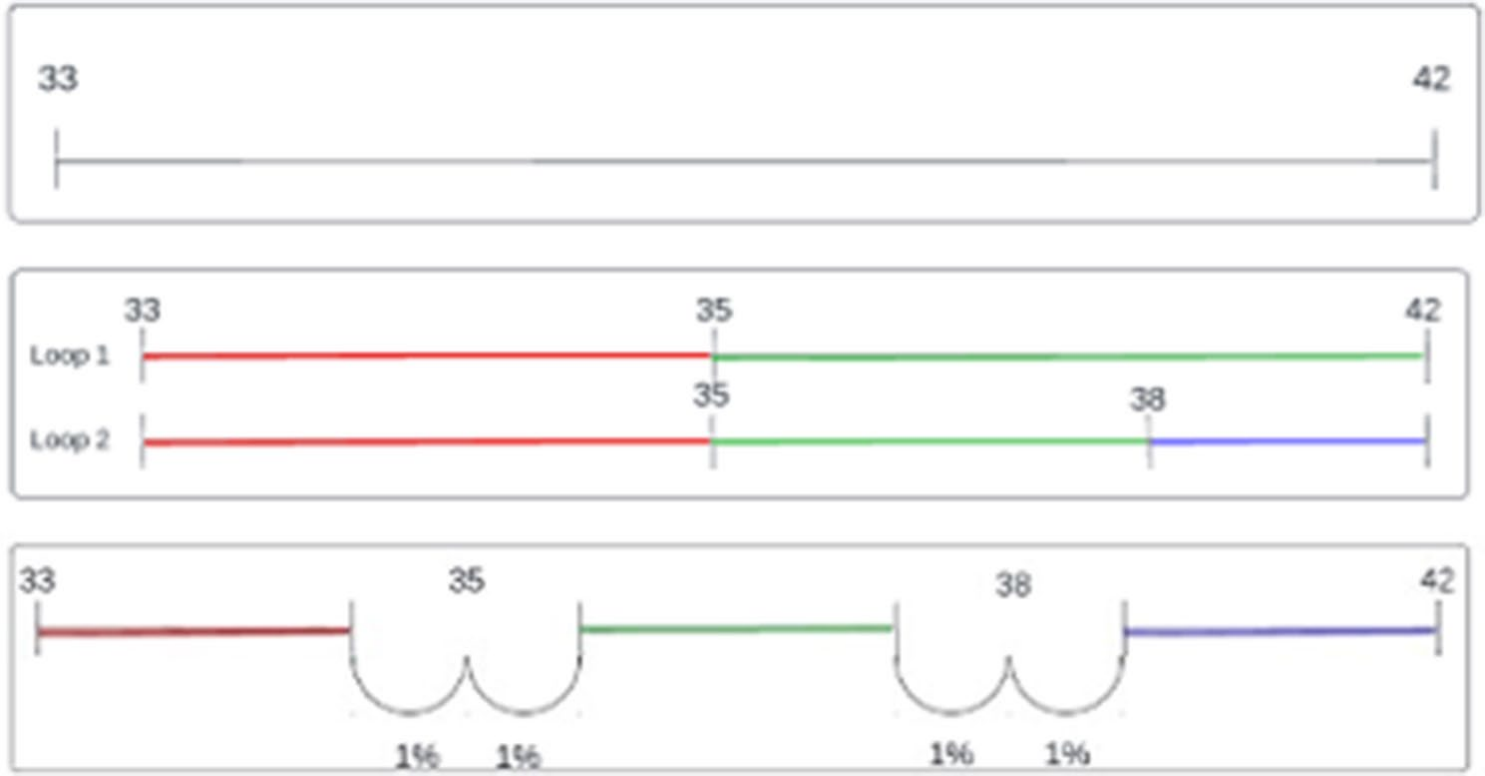
Algorithm process

The first step in this loop is analyzing the subset. The values of the selected continuous column, seen in Fig. 6 , will be partitioned into multiple subsets by defining thresholds. To find these thresholds a function is called, that uses a method defined by Quinlan [47] that extracts the best threshold and its corresponding information gain ratio, by looping through all numbers and finding the best spot to split where the target values are divided the most [47]. In Fig. 6, the identified thresholds of a continuous column containing body temperature are presented. All subsets will be split until there is no longer a subset where its best threshold has a greater information gain ratio than the defined minimum gain ratio, which is presented in Fig. 5.

**Fig. 7:**
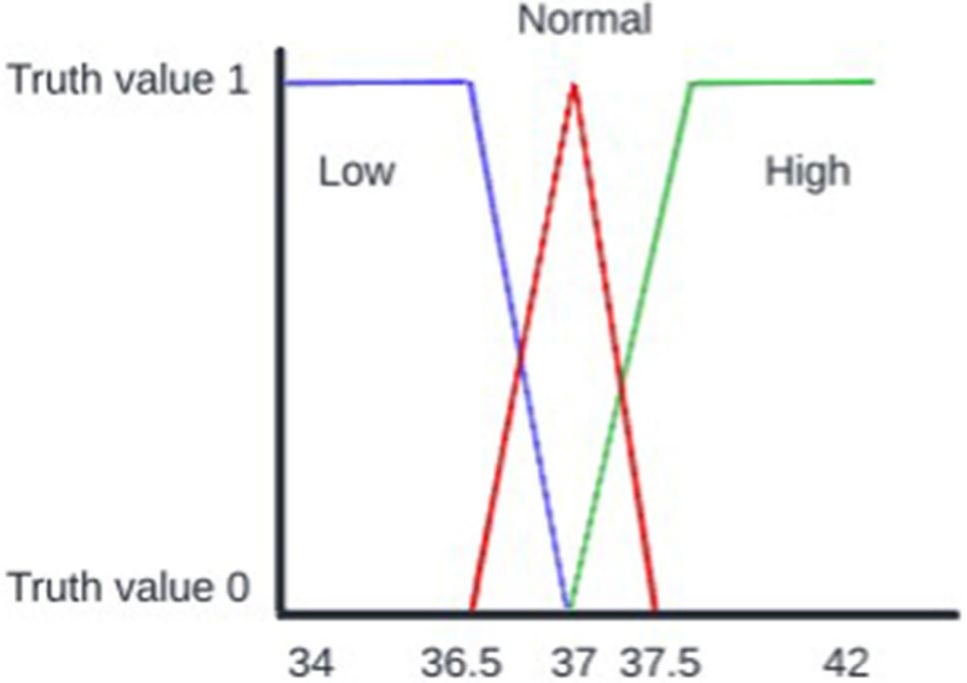
Trapezoidal membership function of fuzzy classifier algorithm

To create the fuzzy aspect, transition periods are implemented. By extending each threshold to a given percentage into each subset it divides, see Fig. 6. This creates a point to create a linear line to distinguish where a particular temperature belongs. After the dataset is split, the transition periods are created to create a fuzzy transition between subsets. These are created by extending the thresholds with a certain given percentage of the subsets adjacent to it, visualized by Figs. 6 and 7.

Membership functions serve as the backbone of the fuzzy logic integrated into our algorithm. These functions are graphical representations that map each element in the input space to a degree of membership between 0 and 1. In simpler terms, they quantify how much a particular data point belongs to a fuzzy set or category (Fig. 7).

Various types of membership functions can be used, such as triangular, trapezoidal, or Gaussian, depending on the specific requirements of the problem at hand [48, 49]. For the purpose of this study, we employ a trapezoidal type of membership function tha t best suits the nature of our healthcare data.

In the context of our fuzzy classifier algorithm, membership functions play a pivotal role in translating continuous values into fuzzy categories. For example, consider a temperature reading of 36.5 °C. The membership function will determine the degree to which this value belongs to different fuzzy sets like “Low,” “Normal,” or “High.” It could indicate that the value is 25% “Low” and 75% “Normal,” thereby providing a more nuanced understanding than a binary classification would. One of the key advantages of using membership functions is their ability to handle uncertainty and ambiguity effectively. In healthcare settings, data can often be imprecise or incomplete. Membership functions allow the algorithm to work with this uncertainty by assigning degrees of membership to fuzzy sets of data, rather than forcing a hard classification like other classification algorithms (e.g., C4.5). This is particularly useful for accommodating the natural variability and complexity often encountered in medical data. In practical terms, these membership functions can be invaluable for healthcare professionals. For instance, when diagnosing a condition that has a range of symptoms with varying degrees of severity, the membership functions can help in making more informed and nuanced decisions. They offer a way to integrate a spectrum of clinical observations and test results into a unified decision-making framework. By incorporating membership functions into our fuzzy classifier algorithm, we aim to provide a tool that is not only accurate but also adaptable to the complexities and uncertainties inherent in healthcare decision-making. The pseudo-code of the fuzzy algorithm can be found in Appendix A.

**Fig. 8:**
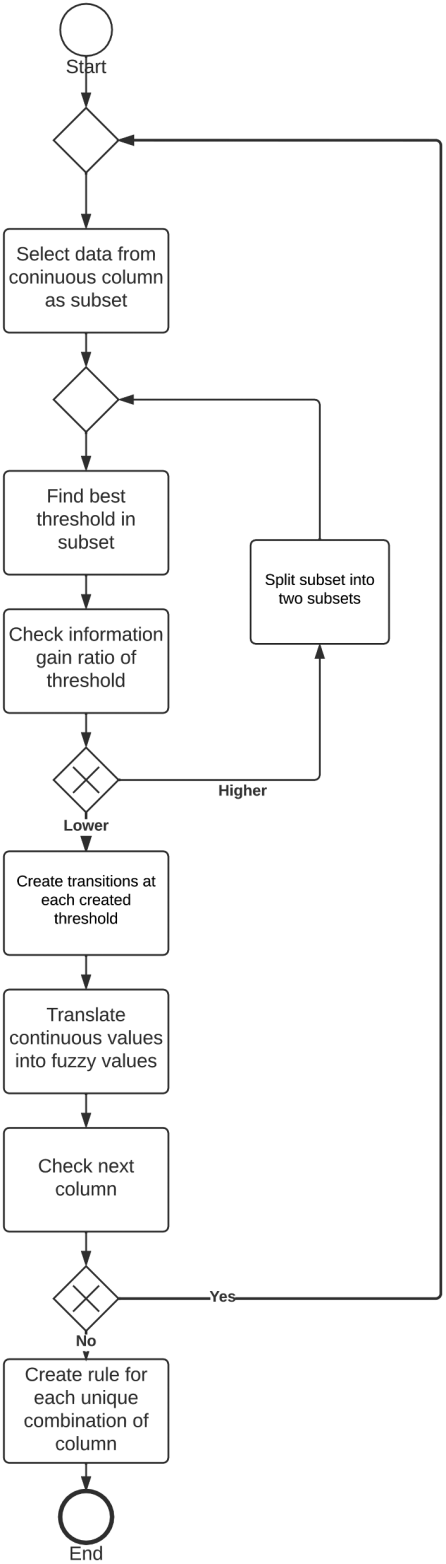
Overall process of algorithm

The overall process of the adapted fuzzy algorithm is presented in Fig. 8 in Business Process Management Notation.

### From dataset to DMN notation

Once the dataset has been partitioned and the fuzzy transition periods have been established, the algorithm proceeds to the crucial phase of rule formation. In this stage, the algorithm generates rules based on each unique combination of variables in the dataset. These rules serve as the foundation for decision-making and are essential for the algorithm’s primary objective discovering and visualizing decisions. The rules are generated by evaluating the degrees of membership assigned to each data point in the fuzzy sets. For example, if a temperature reading of 36.5 °C has a 25% membership in the “Low” category and a 75% membership in the “Normal” category, a rule might be generated that says, “If the temperature is around 36.5°C, then it is Normal”. The confidence levels of each rule are saved, so that subject matter experts can validate the rules on their corresponding confidence levels.

After the rules are formulated, they are translated into an XML format compatible with Decision Model and Notation (DMN) decision tables. DMN is a standard for decision-making and provides a common notation that is easy to understand for both technical and non-technical stakeholders. This translation is vital for ensuring that the algorithm’s output can be readily interpreted and utilized by healthcare professionals such as nurses or physicians or changed and implemented into information systems.

To make the fuzzy values, generated by the algorithm, interpretable by human analysts, an encoding process is applied. This encoding process employs an interchangeable encoder that uses positional terms to represent the fuzzy values. In fuzzy logic, a branch of mathematics and artificial intelligence, sets are often described in terms of degrees of membership. These degrees are typically categorized using specific terms from the field, such as ‘nadir’ for the lowest degree, ‘median’ for a middle degree, and ‘zenith’ for the highest degree of membership in a fuzzy set. However, when communicating these concepts to Subject Matter Experts or those less familiar with the technical jargon, these terms are translated into more universally understood ordinal categories that are context-specific, like ‘Low,’ ‘Normal,’ and ‘High.’ as shown in Table 2. This translation is essential for ensuring that the rules are not only accurate but also easily understandable. It allows healthcare professionals to quickly grasp the implications of the rules without having to delve into the complexities of fuzzy logic or algorithmic computations. The encoding process is particularly important in healthcare settings, where quick and accurate decision-making is often crucial. By translating complex fuzzy values into easily understandable terms, the algorithm makes it easier for healthcare professionals to integrate the outcomes into their decision-making processes. This can be particularly useful in high-stakes environments like emergency rooms or intensive care units, where decisions often must be made rapidly and with incomplete information.

**Table 2:** Conversion from positional to translation term (given a situation of three positional terms)

Positional term	Translation term
Nadir	Low
Median	Normal
Zenith	High

### Decision table normalization

After DMN decision tables are generated, an optional normalization process can enhance their utility and manageability. This step involves identifying and consolidating rules that lead to the same decision outcome, thereby improving the table’s readability and ease of use.

Consider a straightforward example:

1) IF gender is Female and age is 24 and Temperature is Normal THEN Treatment is Treatment B.

2) IF gender is Female and age is 20 and Temperature is Normal THEN Treatment is Treatment B.

These could be consolidated into one rule:

If gender is Female and Age is 20 or 24 and Temperature is Normal THEN Treatment is B.

For a more complex normalization, consider the following rules:

1. IF Blood Type is O and Age is under 30 THEN Treatment is Treatment X.
2. IF Blood Type is O and Age is 30 to 40 THEN Treatment is Treatment X.
3. IF Blood Type is A and Age is under 30 THEN Treatment is Treatment Y.
4. IF Blood Type is A and Age is 30 to 40 THEN Treatment is Treatment Y.

These rules can be restructured into:

1. IF Blood Type is O and Age is under 40 THEN Treatment is Treatment X.
2. IF Blood Type is A and Age is under 40 THEN Treatment is Treatment Y.

This more advanced example shows that even complex decision tables can be simplified through normalization without affecting the outcome or the integrity of the decisions. Importantly, the algorithm ensures that this simplified table remains a faithful representation of the original decision logic. This ensures that all unique rules that are discovered are shown in the final decision table.

## Simulation case

**Fig. 9:**
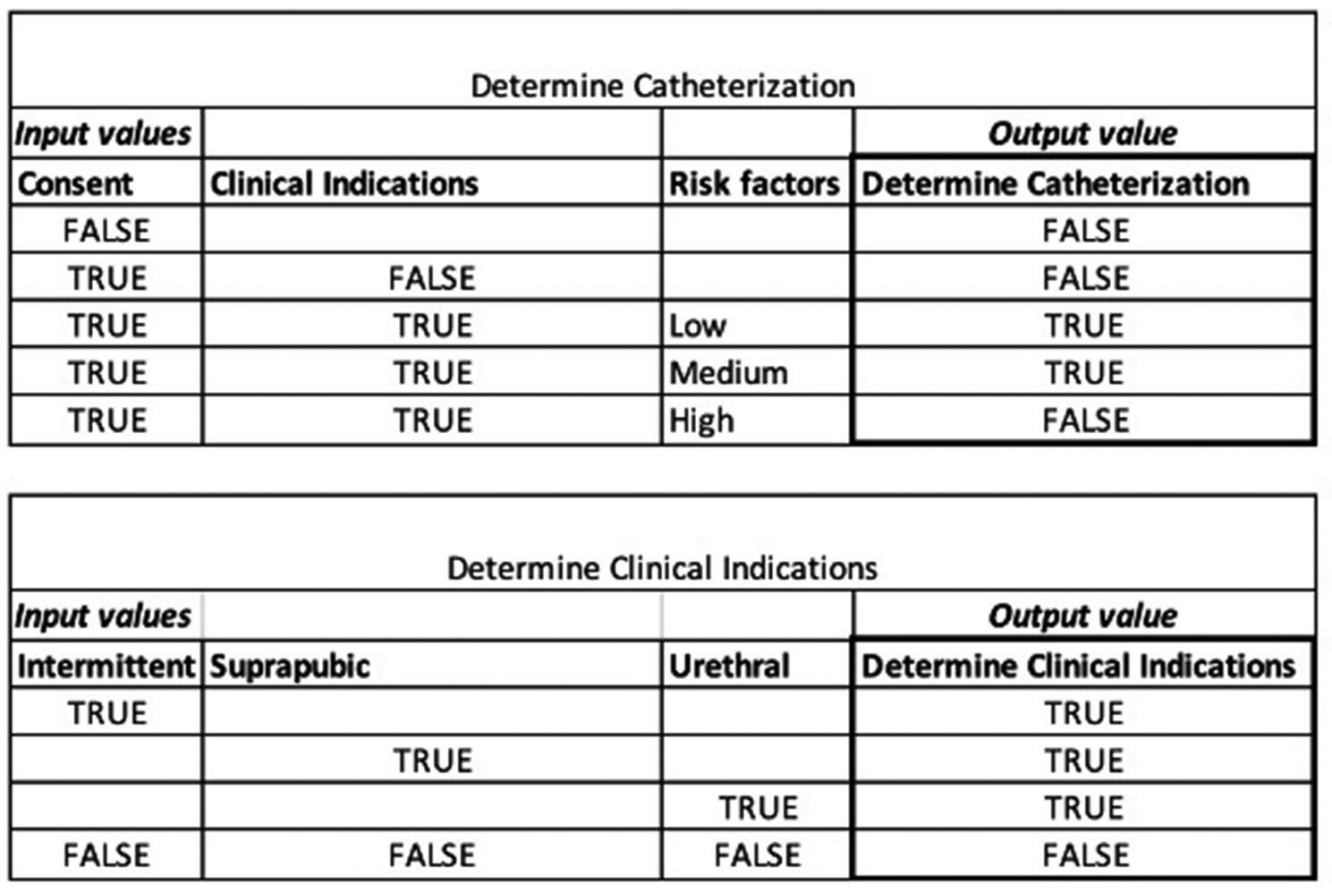
Theoretical model of decision table catheterization

**Fig. 10:**
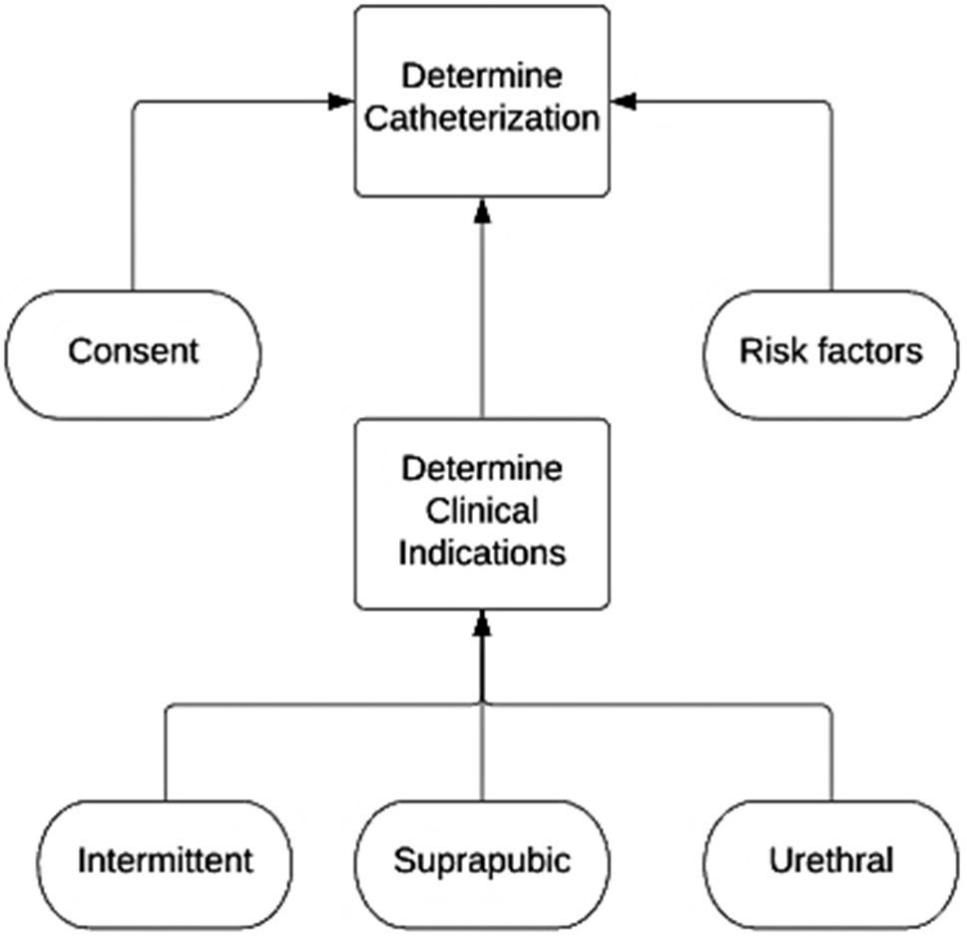
Theoretical model of DRD catheterization

To demonstrate the mechanics of the algorithm we use an authentic case in the healthcare domain consisting of synthetic data. This case is based on the decision made by nurses to insert a catheter. This decision is described in a guideline used by healthcare professionals. A good example would be the guideline created by the Royal College of Nursing [38]. While the guideline consists of multiple steps, we extracted the decision of whether to insert a catheter. This decision is made based on three conditions, (1) Consent, (2) Clinical consideration, and (3) Risk factor. Consent and Clinical consideration are Boolean variables and Risk factor is an integer variable. The Risk factor is interpreted and divided into Low, Medium, and High Risk. The outcome, inserting a catheter, is also a Boolean variable. A theoretical model of both the decision table and a decision requirement diagram (DRD) is created, presented in Figs. 9 and 10.

For the purpose of this simulation study, a synthetic dataset was created to represent a decision log for the clinical decision “Determine Catheterization.” The dataset was generated using a data generator tool^1^ designed to simulate real-world scenarios. This tool allows for the creation of synthetic data that closely mimics the characteristics of actual clinical data, thereby enhancing the validity of the simulation.

After the synthetic dataset was generated, it was followed by validating the data. The validation was conducted by a researcher in the field. The researcher ensured that the data represented the variables and conditions typically encountered in clinical settings for catheterization decisions.

**Fig. 11:**
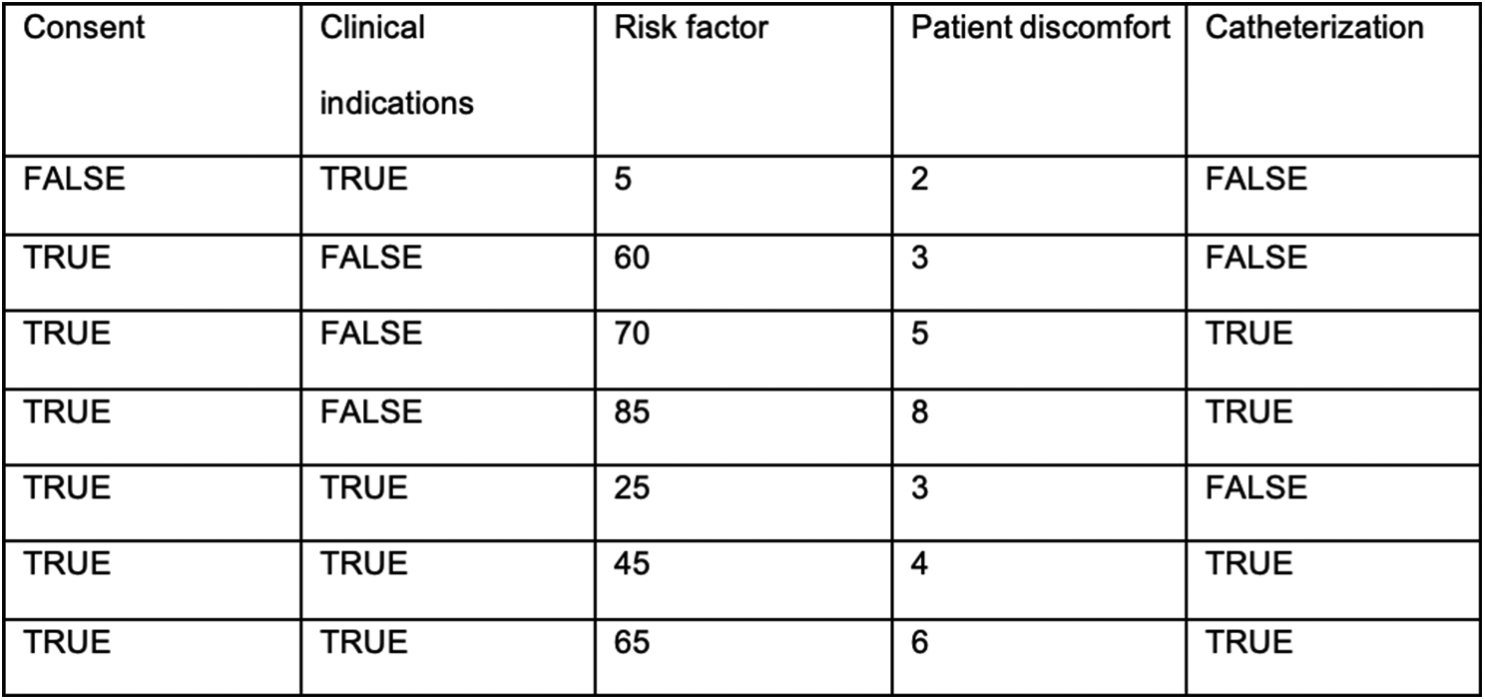
Excerpt of decision log

The synthetic dataset consists of 1,000 rows, each representing a clinical scenario related to catheterization decisions. The dataset is complete, with no missing values in any of the columns. This completeness is intentional to focus the simulation on the decision-making processes rather than data quality challenges. In this case, we deviated from the theoretical model by adding another input value that is influencing the decision, namely patient discomfort. Patient discomfort is an integer between one and ten, where 1 is no discomfort and 10 is the most discomfort a patient has experienced. It’s worth noting that while typical decision logs might include ‘Timestamp’ and ‘ID’ columns to track the timing and identity of each decision, these columns were deliberately excluded from the synthetic dataset used in this simulation. The rationale for this exclusion is that these columns are not pertinent to the decision-discovery process that is the focus of this study. A cutout of the first five rows of the decision log is presented in Fig. 11.

**Fig. 12:**
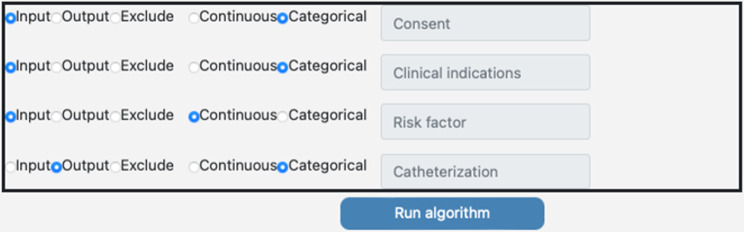
Assign meta-data to columns (screenshot from application to run the algorithm)

The risk factor is shown as an integer in the decision log. The algorithm will transform the numerical risk factor to a low, medium, or high risk using a fuzzy classifier. The algorithm is written in Python and runs in a docker container^2^. The decision log is converted to a comma separated value (CSV) file to load the decision log into the implementation of the fuzzy classifier algorithm. The next step is to assign the correct meta-data to the columns, as seen in Fig. 12. In this case, the columns Consent, Clinical Indications, and Catheterization contain categorical values (boolean) and the variables Risk factor and patient discomfort contain continuous values (integer).

**Fig. 13:**
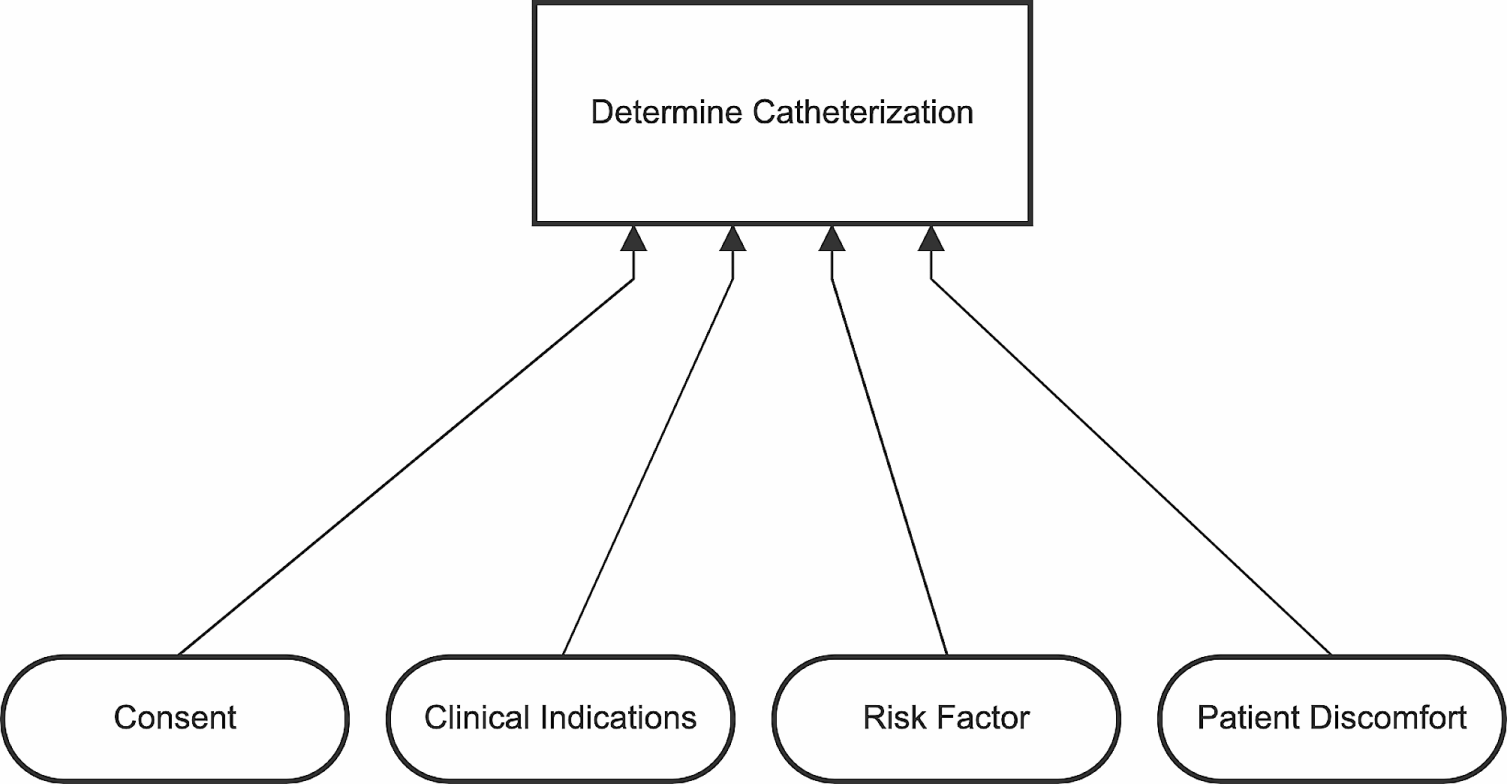
screenshot of the DRD output of a decision log

**Fig. 14:**
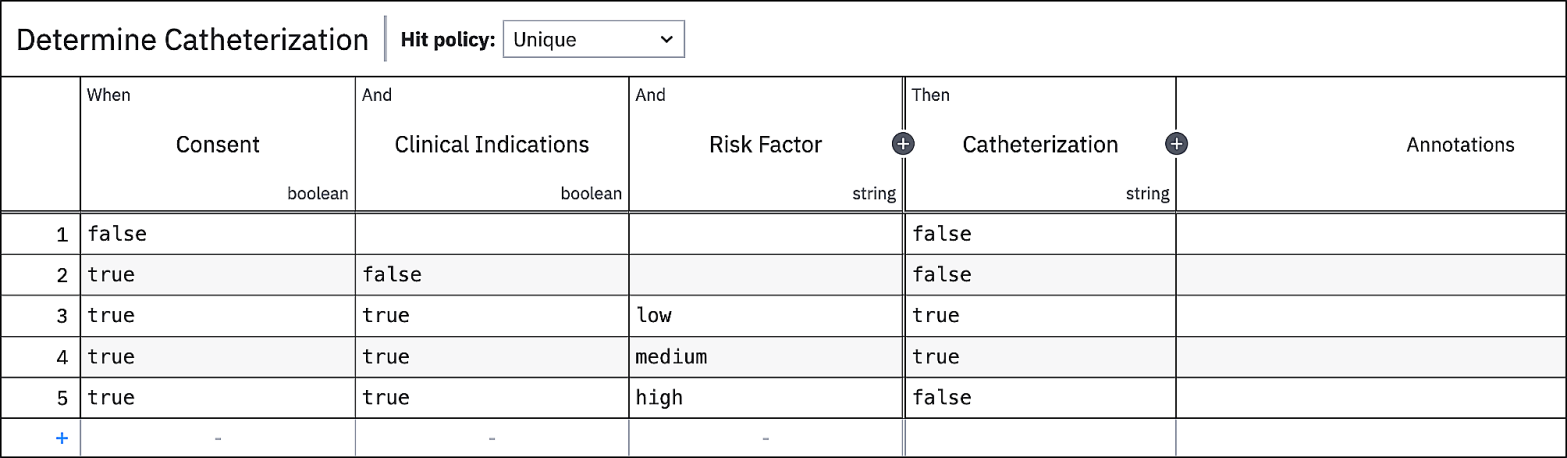
Decision table output of decision Determine Catheterization (screenshot)

The algorithm starts running after this step. In this simulation, the runtime of the algorithm was 2.18 s. Figure 13 presents the normalized decision table that is created from the decision log. The found decision rules are represented here. Figure 14 presents a DRD with the input values and the decision.

**Fig. 15:**
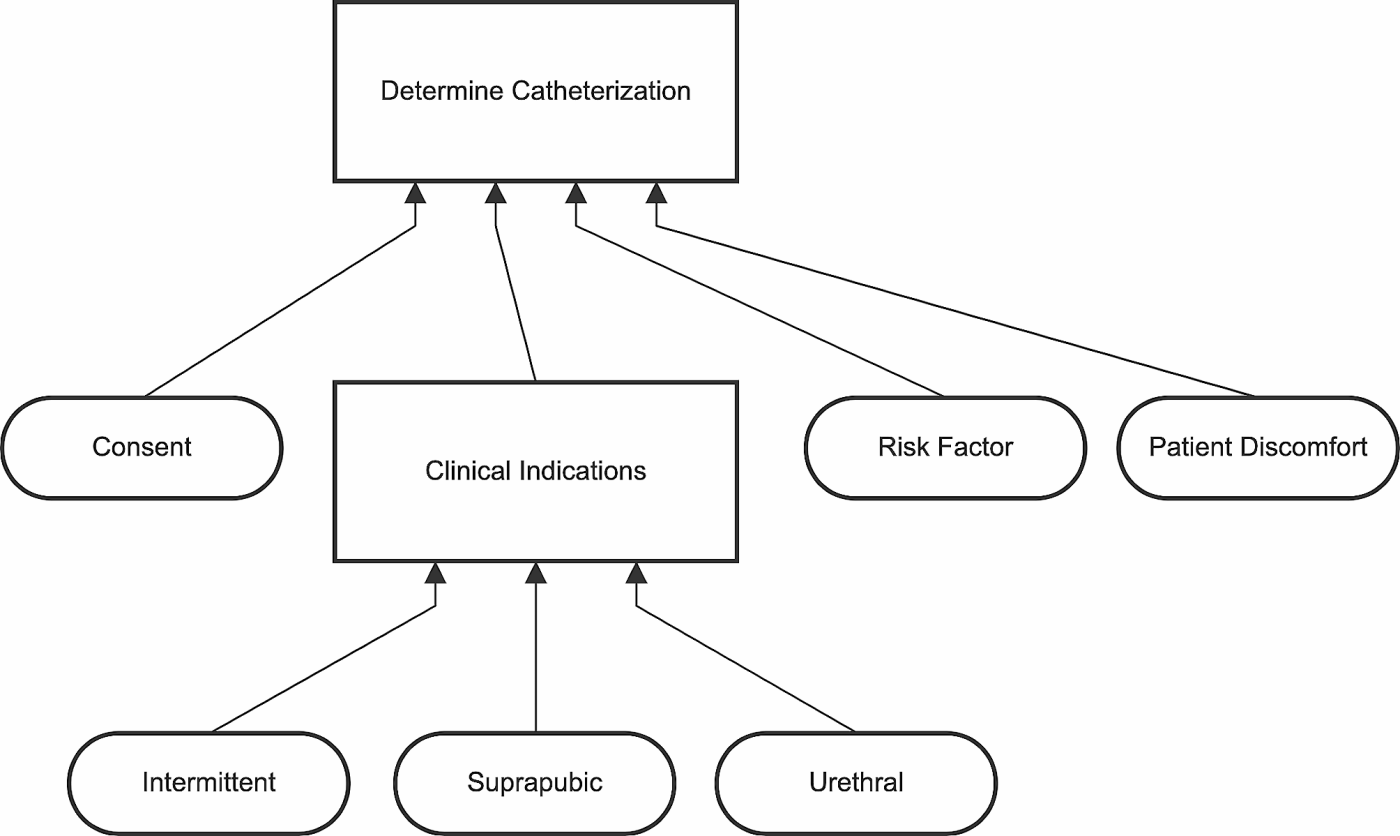
DRD of combined decision logs (screenshot)

**Fig. 16:**
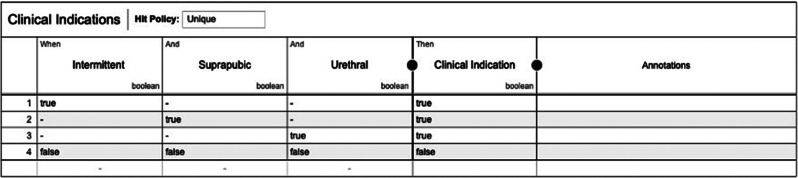
Decision table of decision determine clinical indications (screenshot)

To simulate a second decision, another decision log, consisting of data for the decision Determine Clinical Indications is run through the algorithm. The decision Determine Clinical Indications consists of three input variables, namely: Intermittent, Suprapubic, and Urethral which are all Boolean variables. The runtime of the second decision log was 1.68 s. Figures 15 and 16 present the model that is created by combining the first and second decision log. It contains a normalized decision table of the second decision and a DRD presenting the dependency between Catheterization and clinical indications. In this simulation first the Determine Catheterization is executed, but the datasets can be executed in any particular order.

While the protocol for inserting a catheter is followed, another input variable is used in practice to make the decision. By using the algorithm the decision model, including the new variable is found and visualized.

## Discussion

In this paper, we propose an algorithmic solution to discover business decisions and business logic in a healthcare context. The developed algorithm is based on a fuzzy decision tree classifier. Earlier approaches, for example, Bazhenova et al. [45], proposed an algorithm based on a neuro fuzzy classifier to discover business decisions and underlying business logic in event logs. However, neuro fuzzy classifiers are black-boxes and therefore not transparent and explainable. For the specific case of discovering decisions in healthcare, black box algorithms are not preferable. For example, the algorithm is used to give feedback on a clinical decision support system or a physician. Both algorithms could discover the decisions from structured data and visualize them, but the neuro fuzzy classifier is not explainable. The fuzzy algorithm proposed in this paper is explainable and transparent by nature, which is one of the recurring challenges in developing trust for using algorithms and CDSSs [50].

One of the algorithm’s strengths lies in its generalizability. The algorithm is designed to work with a specific input format that closely resembles the output log files commonly generated by Clinical Decision Support Systems (CDSSs) or Electronic Health Records (EHRs). This design choice was intentional and serves to enhance the algorithm’s applicability across multiple healthcare settings and systems. Because many healthcare institutions use CDSSs or EHRs that produce log files in a similar format due to the fact of more standardization of data interoperability protocols e.g., HL7 [51], the algorithm can be readily applied without requiring extensive data transformation or pre-processing except a subject matter expert to check the relevant data columns. However, it’s worth noting that this strength is a double-edged sword. While the strict input format allows for broad applicability across similar datasets, it also imposes limitations on the types of data the algorithm can process. Datasets that do not conform to this specific format would require additional manipulation, which could introduce errors or inconsistencies. Therefore, while the algorithm’s generalizability is an advantage, it is not without constraints that users should be aware of.

The output of this algorithm is a Decision Requirements Diagram (DRD) and the underlying decision table(s) according to the DMN standard [52]. We argue that there are multiple reasons to visualize decisions in this standard. The first reason is that this standard is widely adopted in decision management systems and is gaining attention in the healthcare sector for modeling decisions for healthcare professionals due to the visualization of decisions [53–55]. The DMN standard is understandable for healthcare professionals, e.g., nurses, but the decisions can be modeled by data or information analysts. An advantage of this standard is that the discovered decisions and decision tables can be imported into decision management systems to, e.g., validate and test the identified decisions or potentially directly used in a CDSS as decision rules. For example, during the COVID-19 pandemic, several decision models created in DMN were published to both the public and hospitals [54]. Another reason is that this standard supports integration with the Business Process Model and Notation (BPMN) [56]. Decisions are separated from process models for better maintaining the decisions and business logic since these rules change more often compared to the process model. Both standards are complementary to each other. This possible entanglement provides another advantage as process mining and decision mining can be combined. By using process mining algorithms to find process steps, decision mining algorithms can be used to find the decisions and underlying business logic.

From a practical perspective, the discovery of decisions out of data can be used for improvements and evaluation of decisions taken in practice. For example, in the Netherlands, a quality manager is assigned to a nursing department. The quality manager is responsible for maintaining the quality of the team and is in most cases a nurse. The output of this algorithm can be used to confirm the way of working with the protocols and guidelines for that department.

The algorithm itself has some technical limitations. As the algorithm is written in Python it cannot deal with heterogenous datasets and is therefore, at the moment limited to fixed datatypes. Another limitation is that the performance of the algorithm during execution becomes exponentially slower the larger the dataset is, both in the number of columns as well as in number of rows. Due to this fact, we limited the number of input data columns to ten. For instance, if both the number of input values and the number of rows are near the limits, the execution time exceeds twenty hours. Although the algorithm’s performance could be enhanced by using other programming languages like Java or C, we argue that when the algorithm is run only once or twice a year, performance becomes less important compared to the ease of use offered by Python.

The last technical limitation is that the fuzzy algorithm, at the moment, can handle a maximum of five positional terms, thus only recognizing five categories while, potentially, more categories could be present in the data.

This limitation refers to the number of distinct categories that can be recognized for each variable, not the total across all variables in the dataset. This constraint is pivotal in maintaining algorithmic efficiency and clarity in decision-making processes. However, it can potentially raise questions about the algorithm’s suitability for complex clinical scenarios involving more than five categories per variable. While scarce, to accommodate more than five categories in the algorithm, an adaptation can be made within the source code. This could, for example be necessary due to the number of categories in protocols present, however this increase of categories drastically impacts the efficiency of the algorithm.

From a practical viewpoint, we can identify a limitation that the algorithm, at the moment, heavily depends on subject matter experts in both the data preparation as well as the validation phase. For the data preparation part, the subject matter expert must select or check relevant columns in a large dataset that can be transformed into a decision log as the export of different systems differs. The algorithm can be used on a wide set of data, if the data structure is the same. Therefore, validation is important. For example, concerning the handling of outliers in data. The algorithm considers outliers as part of a spectrum rather than discrete exceptions. By applying fuzzy sets, these occurrences are integrated into the decision-making process with a degree of membership, acknowledging their existence without allowing them to skew the overall results. This method is particularly beneficial in healthcare, where every data point, no matter how atypical, could be crucial. Thus, the algorithm recognizes and incorporates the unique characteristics of outliers, ensuring a comprehensive analysis. However, we suggest that the identified decisions must be validated by subject-matter experts, such as nurses or physicians, to be able to check the decisions and identify anomalies.

## Conclusion & future work

In this study, we answer the following research question: *How can the fuzzy algorithm be adapted to discover business decisions and business logic in a healthcare context?* We propose a novel approach to discover decisions from structured data using a fuzzy decision tree classifier and visualize them using the DMN standard. From a practical viewpoint, the visualization supports the understanding of the decision-making process and gives feedback to a physician or nurse. By using DMN as visualization it is both comprehensible for the physician or nurse as well as technically interchangeable between CDSSs. From a theoretical viewpoint, the proposed algorithm discovers decisions using structured data. Future work could be focused on testing the algorithm on more diverse datasets. Another study could be the comparison between different algorithms to see which algorithm has the best results on a specific dataset, which should be checked against subject matter experts. In the end, the algorithm could be tested with datasets from different healthcare organizations that use the same protocol or guideline to find similarities and deviations between the healthcare organizations.

### Electronic supplementary material

Below is the link to the electronic supplementary material.

Supplementary Material 1

## Data Availability

The datasets generated and/or analyzed during the current study are available in the GitHub DecisionMiningFuzzy repository, https://github.com/HU-DIPO/DecisionMiningFuzzy.

## Footnotes

1 https://www.mockaroo.com/.

2 https://github.com/HU-DIPO/DecisionMiningFuzzy.
