# Supplementary material for: Decision discovery using clinical decision support system decision log data for supporting the nurse decision-making process
Source: BMC Med Inform Decis Mak. 2024 Apr 18;24:100. doi: 10.1186/s12911-024-02486-3 (PMC11025262; doi:10.1186/s12911-024-02486-3)
Supplement: Supplementary file 1 — Supplementary Material 1 [file 12911_2024_2486_MOESM1_ESM.docx]

**Appendix A:**

| 1 | Fuzzyclassifier (dataset, continuous cols, minimum gain ratio) | | | |  |  |  |
| --- | --- | --- | --- | --- | --- | --- | --- |
| 2 | INPUT: dataset, list of continuous columns and a number of what the minimum gain ratio needs to be. | | | | | | |
| 3 |  |  |  |  |  |  |  |
| 4 | rules = () |  |  |  |  |  |  |
| 5 |  |  |  |  |  |  |  |
| 6 | **for each** column idx in continuous cols | | |  |  |  |  |
| 7 |  | split up each continuous column in thresholds where gain ratio > minimum gain ratio | | | | | |
| 8 |  | create transition periode between thresholds. | | |  |  |  |
| 9 |  | Replace value with it corresponding threshold index | | | |  |  |
| 10 |  |  |  |  |  |  |  |
| 11 | combinations is all the unique input combinations | | | |  |  |  |
| 12 | **for each** combination in combinations | | |  |  |  |  |
| 13 |  | mask is where row in x == combination | | |  |  |  |
| 14 |  | results is where misk is applied to y | | |  |  |  |
| 15 |  | rules [combination] = Modus (combination) | | |  |  |  |

FuzzyClassifier Transforms continuous values into Fuzzyvalues. Where the continuous values split into Fuzzyvalues based on the gain ratio (if its higher then minimum gain ratio). Then creates a dictionary where the key is the decision combination. And the value is the Modus of the results of the decision combination.
